# OncoCTMiner: streamlining precision oncology trial matching via molecular profile analysis

**DOI:** 10.1093/database/baad077

**Published:** 2023-11-04

**Authors:** Quan Xu, Yueyue Liu, Dawei Sun, Xiaoqian Huang, Feihong Li, JinCheng Zhai, Yang Li, Qiming Zhou, Niansong Qian, Beifang Niu

**Affiliations:** Department of Bioinformatics, Beijing ChosenMed Clinical Laboratory Co. Ltd., Jinghai Industrial Park, 156 Jinghai 4th Road, Economic and Technological Development Area, Beijing 100176, China; Research and Development Center, ChosenMed Technology (Zhejiang) Co. Ltd., Room 101, Building 8, Jincheng International Science and Technology City, No. 26 Zhenxing East Road, Linping District, Hangzhou, 311103, China; Department of Bioinformatics, Beijing ChosenMed Clinical Laboratory Co. Ltd., Jinghai Industrial Park, 156 Jinghai 4th Road, Economic and Technological Development Area, Beijing 100176, China; Department of Bioinformatics, Beijing ChosenMed Clinical Laboratory Co. Ltd., Jinghai Industrial Park, 156 Jinghai 4th Road, Economic and Technological Development Area, Beijing 100176, China; Research and Development Center, ChosenMed Technology (Zhejiang) Co. Ltd., Room 101, Building 8, Jincheng International Science and Technology City, No. 26 Zhenxing East Road, Linping District, Hangzhou, 311103, China; Department of Bioinformatics, Beijing ChosenMed Clinical Laboratory Co. Ltd., Jinghai Industrial Park, 156 Jinghai 4th Road, Economic and Technological Development Area, Beijing 100176, China; Department of Bioinformatics, Beijing ChosenMed Clinical Laboratory Co. Ltd., Jinghai Industrial Park, 156 Jinghai 4th Road, Economic and Technological Development Area, Beijing 100176, China; Department of Bioinformatics, Beijing ChosenMed Clinical Laboratory Co. Ltd., Jinghai Industrial Park, 156 Jinghai 4th Road, Economic and Technological Development Area, Beijing 100176, China; Beijing International Center for Mathematical Research, Peking University, No. 5 Yiheyuan Road Haidian District, Beijing 100871, China; Chongqing Research Institute of Big Data, Peking University, Chongqing 401333, China; Department of Bioinformatics, Beijing ChosenMed Clinical Laboratory Co. Ltd., Jinghai Industrial Park, 156 Jinghai 4th Road, Economic and Technological Development Area, Beijing 100176, China; Research and Development Center, ChosenMed Technology (Zhejiang) Co. Ltd., Room 101, Building 8, Jincheng International Science and Technology City, No. 26 Zhenxing East Road, Linping District, Hangzhou, 311103, China; Department of Oncology, Senior Department of Respiratory and Critical Care Medicine, The Eighth Medical Center of Chinese PLA General Hospital, No.17 A Heishanhu Road, Haidian District, Beijing 100853, China; Computer Network Information Center, Chinese Academy of Sciences, Beijing 100190, China; University of Chinese Academy of Sciences, Beijing 100190, China

## Abstract

By establishing omics sequencing of patient tumors as a crucial element in cancer treatment, the extensive implementation of precision oncology necessitates effective and prompt execution of clinical studies for approving molecular-targeted therapies. However, the substantial volume of patient sequencing data, combined with strict clinical trial criteria, increasingly complicates the process of matching patients to precision oncology studies. To streamline enrollment in these studies, we developed OncoCTMiner, an automated pre-screening platform for molecular cancer clinical trials. Through manual tagging of eligibility criteria for 2227 oncology trials, we identified key bio-concepts such as cancer types, genes, alterations, drugs, biomarkers and therapies. Utilizing this manually annotated corpus along with open-source biomedical natural language processing tools, we trained multiple named entity recognition models specifically designed for precision oncology trials. These models analyzed 460 952 clinical trials, revealing 8.15 million precision medicine concepts, 9.32 million entity-criteria-trial triplets and a comprehensive precision oncology eligibility criteria database. Most significantly, we developed a patient-trial matching system based on cancer patients’ clinical and genetic profiles, which can seamlessly integrate with the omics data analysis platform. This system expedites the pre-screening process for potentially suitable precision oncology trials, offering patients swifter access to promising treatment options.

**Database URL** https://oncoctminer.chosenmedinfo.com

## Background

Molecular profiling of patient tumors has become a critical component of cancer treatment, owing to the identification of novel therapeutic targets and the growing use of precision medicine-based therapies. Individualized cancer therapy based on genetic markers can improve response rates and extend progression-free survival (1). Despite the potential therapeutic benefits of many targeted and immunotherapies, they are still in the clinical trial stage (2), and there is a need for more participants in innovative precision oncology drug trials to enhance cancer therapy (3). However, only approximately 8% of cancer patients participate in clinical trials (3, 4). Despite increased genomic profiling, only 10–15% of individuals with actionable mutations in their genomic profiles participate in precision oncology clinical trials (5–9). Low clinical trial participation can be attributed to several factors, such as a lack of physician knowledge regarding acceptable studies, patient performance status and patient attitudes and financial concerns (10–12).

Connecting patient genetic data to precision oncology trial eligibility criteria presents another challenge in the recruitment of patients to clinical trials (13, 14). Without sophisticated trial-matching systems, physicians must navigate hundreds of rapidly evolving active trials to determine the few that may be suitable for an individual patient (15, 16). Even oncologists at top cancer centers have expressed doubts about their genetic expertise (17). While tumor next-generation sequencing testing facilities often provide trial suggestions for patients based on their clinical and genomic profile (18), maintaining these databases can also be time-consuming. From a clinical investigator’s perspective, the average time spent on patient enrollment from initial identification to final enrollment was estimated to be 3.4 to 8.8 hours and $129 to $336, respectively (19). More effective and streamlined solutions are needed for patient-trial matching.

**Figure 1.**
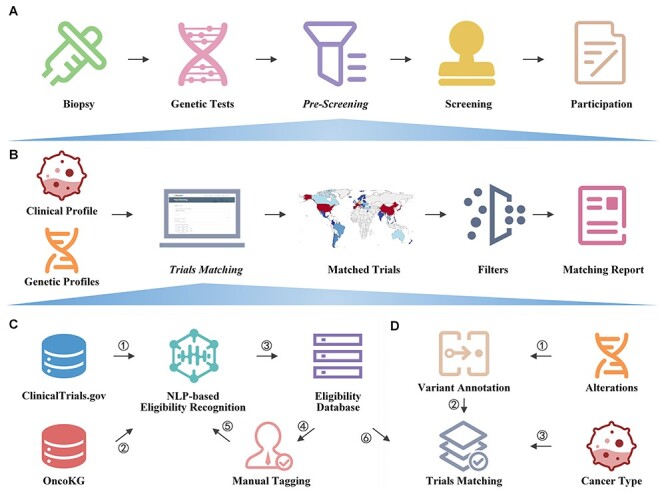
Overview of OncoCTMiner.

ClinicalTrials.gov (https://clinicaltrials.gov/) is the most widely used clinical trial database, containing information on clinical trials conducted in 221 countries. However, its structured data are insufficient for automated patient trial matching, especially when eligibility requirements involve genetic information. Various clinical trial knowledge bases have been created by the precision oncology community, such as My Cancer Genome (20), which are based on databases like ClinicalTrials.gov but only allow searching for trial data rather than automated trial matching. While systems like MatchMiner (14), OCTANE (21), Criteria2Query (22), and the Stanford Patient Eligibility Screening Algorithm (23) provide patient-trial matching functionalities, they are generally proprietary and difficult to implement by other institutions (Supplementary File 1). To address the gap in patient-trial matching, we created OncoCTMiner, an open and free platform that enables real-time clinical trial matching of tumor genetic testing samples for precision oncology clinical trials (Figure 1). This platform is expected to facilitate patient recruitment for precision oncology clinical trials.

A) OncoCTMiner’s role in precision oncology trial enrollment. B) OncoCTMiner takes clinical and genetic profiles as inputs and utilizes a trial matching and filtering system to generate a report of matched trials. C) Strategy for building the clinical trial eligibility criteria database. D) Automatic matching strategy for genomics-driven oncology trials.

The aim of this paper is to 1) outline the OncoCTMiner workflow; 2) describe how we mine and screen precision oncology clinical trials; 3) explain how we construct a database of precision oncology clinical trial eligibility and search for trials in it; 4) illustrate how we use an automated patient-trial matching platform to pre-screen potentially suitable clinical trials using genetic sequencing results and clinical information of tumor patients. It is expected that this platform will greatly assist clinicians in swiftly and accurately pre-screening precision oncology studies for their tumor patients in the future.

## Implementation

### Text mining

#### Data loader

**Figure 2.**
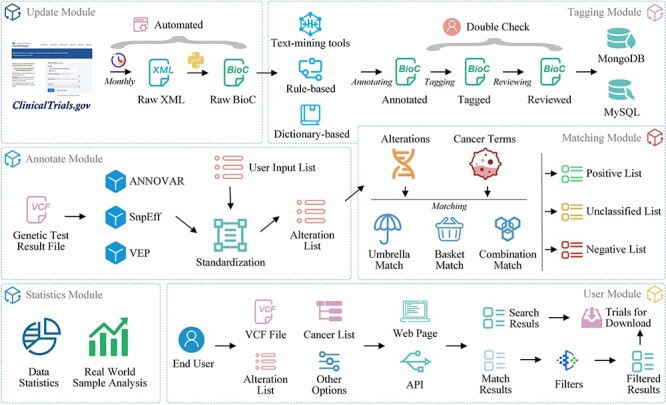
OncoCTMiner modules.

ClinicalTrials.gov is a widely used database providing comprehensive information on clinical trials for both the general public and healthcare professionals (24). To enhance interoperability and facilitate future data processing and exchange, we downloaded the ZIP file containing all study records in extensible markup language format from ClinicalTrials.gov and converted them to BioC-JSON format (25) (Figure 2, update module).

OncoCTMiner consists of several modules: 1) The update module downloads XML-formatted trial data and parses them into BioC-JSON format on a monthly basis; 2) The tagging module identifies bio-concepts using NLP-based tools with double-checking by biomedical professionals to construct the clinical trial eligibility database; 3) The annotation module assists in annotating genetic alterations detected from patient tumors; 4) The matching module utilizes cancer terms and the annotated alterations as key criteria for clinical trial matching; 5) The statistics module provides various statistical analyses of the clinical trial data; and 6) the user module allows users to interact with the system and perform various tasks.

#### Manual tagging

**Figure 3.**
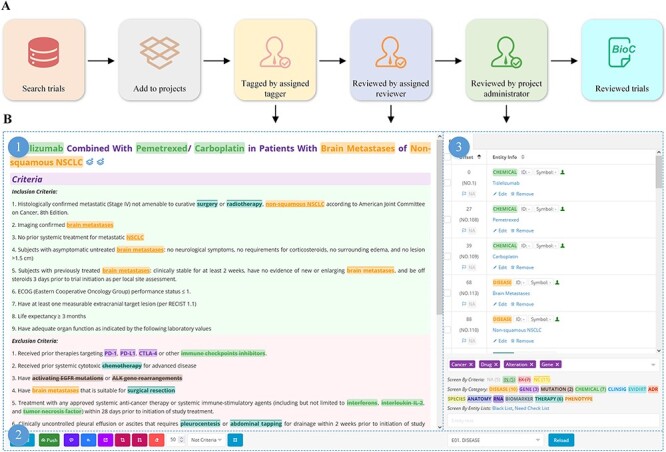
Manual tagging of clinical trials.

We developed a platform for tagging clinical trials based on our previous work (26) (Figure 3). Oncology trials involving gene, alteration, and drug entities are searched, screened, selected and added to a list of pre-designed tagging projects, followed by double-checking by team members (Figure 3A–3C). To ensure that individual annotators have a consistent reference standard and that identified bio-concepts are of high quality, we established a standard processing procedure (Supplementary File 2) for tagging entities.

A) Workflow for manual tagging of clinical trials; B) overview of the trial tagging page: 1) trial details with highlighted bio-concepts, 2) the manual tagging tools bar, and 3) real-time presentation of entity information.

OncoCTMiner aims to establish a comprehensive database of eligibility criteria for oncology trials and connect patients with suitable trials through a search engine and automated matching system. We use the ‘minimization’ principle for entity recognition to improve standardization, for example, dividing ‘HER2-positive breast cancer’ into an alteration and a cancer, and subdividing ‘NSCLC with KEAP1, NFE2L2 and/or STK11 mutation’ into a cancer type, three genes and an alteration, which further normalized into ‘KEAP1:mutation’, ‘NFE2L2:mutation’ and ‘STK11:mutation’.

OncoCTMiner distinguishes itself from comparable systems by not only tokenizing and normalizing biomedical concepts but also determining whether an entity is a recruitment condition for a given clinical trial and its classification based on context. Eligibility criteria are classified into three types: ‘not criteria’ (NC), ‘inclusion criteria’ (inclusion) and ‘exclusion criteria’ (exclusion). Entities outside the eligibility criteria section are categorized as ‘not available’ (NA) since their context cannot be used to evaluate eligibility criteria. We apply the ‘loose inclusion, tight exclusion’ principle to minimize the false negative rate during trial pre-screening while allowing for a relatively high false positive rate to facilitate further manual review of the pre-screened trial list.

#### Entity recognition and standardization

The system identified six categories of biological entities: disease/cancer, genes, alterations, chemicals/drugs, biomarkers and therapies. DNorm (27), GNormPlus (28), tmVar2.0 (29) and tmChem (30) were used to mine disease, genes, alterations and chemical entities, respectively. Biomarkers are indicators discovered by genetic testing or immunohistochemistry that predict the efficacy of specific treatment regimens, such as TMB, MSI and mismatch repair (MMR). Therapies refer to non-drug treatments, including drug treatment categories like ‘chemotherapy’ and ‘immunotherapy’. We constructed two terminologies for the recognition of biomarkers (https://oncoctminer.chosenmedinfo.com/assets/xlsx/dict_biomarker.xlsx) and therapy entities (https://oncoctminer.chosenmedinfo.com/assets/xlsx/dict_therapy.xlsx) using a dictionary-based strategy.

Most entity annotation software matches recognized entities to commonly used databases. For instance, GNormPlus maps annotated genes/proteins to National Center for Biotechnology Information Gene identifiers, tmVar2.0 maps annotated variations to dbSNP RS identifiers, and DNorm and tmChem map annotated diseases/cancers and compounds/drugs, respectively, to Medical Subject Headings (MeSH) (https://www.ncbi.nlm.nih.gov/mesh/) identifiers. However, these standard identifiers do not cover all identified entities, requiring full standardization to facilitate later clinical trial retrieval and matching. We merged and built corresponding term sets for various entity types from several terminology and ontology databases, including OncoTree (31), DiseaseOntology (32), National Cancer Institute Thesaurus (https://ncit.nci.nih.gov/ncitbrowser/start.jsf) and MeSH. We gathered 55 558 cancer entries and established synonym connections or father-child relationships between each item, creating a unique cancer ontology named OncoOntoC (https://oncoctminer.chosenmedinfo.com/assets/xlsx/oncoontoc.xlsx). Using these terminologies or ontologies, we normalized all entities and mapped all synonymous terms to the same standard terms

#### Updates and archive

Clinical trial enrollment status or enrollment criteria may be updated at any time. To ensure the system’s timeliness, we update the trial database monthly, adding new clinical trials as they become available and updating existing trials as their content changes. This guarantees that users always have access to the most up-to-date clinical trial information. Historical versions of clinical trial data, particularly manually annotated data, will be archived as a corpus. As more data are gathered in the future, the entity recognition model will be fine-tuned and recognition efficiency will be constantly enhanced.

### Trials matching

**Figure 4.**
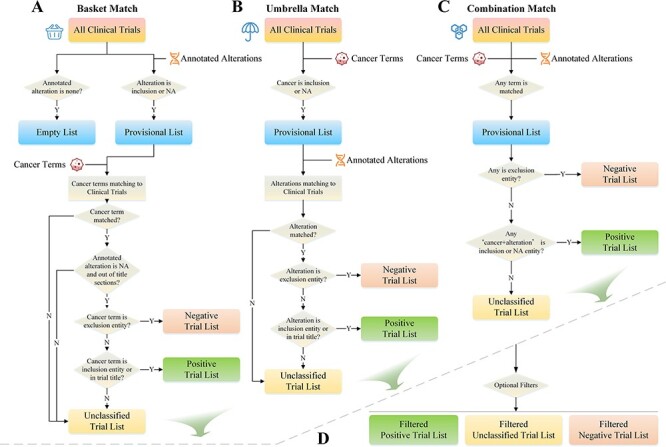
Trials matching strategies.

OncoCTMiner automates clinical trial matching based on the clinical and genetic profiles of tumor patients. Users are prompted to provide clinical data and variant detection results, which are then automatically annotated and matched against the eligibility criteria database. The variant annotation process involves identifying all detected variations in the user-provided variant call format (VCF) format data and mapping them to the standard entry of alterations. For trial matching, the system takes the cancer type selected by the user and the standard alteration terms as input and matches them against clinical trials in the eligibility database (Figure 4).

A) Basket match prioritizes alterations as the primary matching condition; B) umbrella match prioritizes cancer type as the primary matching condition; C) combination match combines multiple matching conditions for more precise matching; D) trial list filter allows users to filter and narrow down the list of matched clinical trials based on various criteria.

#### Alteration annotation

The user-uploaded VCF file undergoes annotation by three software programs, VEP (33), ANNOVAR (34) and SnpEff (35), at the back end of the system. The annotation results are then merged and mapped to the standard entries of alterations. Variation annotation not only matches specific mutations but also determines the type of variation that the mutation belongs to. For example, the mutation ‘EGFR p.L858R’ can match not only ‘EGFR:L858R’ but also ‘EGFR:Activating mutations’, ‘EGFR:exon21mut’, and ‘EGFR:Mutations’ (18). This increases the positive matching rate of clinical trials standardized on these mutations in the system, reduces the chance of missing relevant trials, and offers more hope to patients.

#### Trials matching and screening

To match clinical trials, the system utilizes the cancer types and alteration lists selected by the user as fundamental requirements. Three matching modes are provided: basket, umbrella and combination match. Basket matching selects clinical trials that use variations as inclusion criteria (or NA) as the preferred conditions. These trials are then matched with cancer terms and categorized into negative, positive and unclassified trial lists (Figure 4A). Umbrella matching is similar to basket matching but with cancer and alteration listed as matching conditions in reverse order (Figure 4B). The goal of combination matching is to match both types of entries to the trials simultaneously. If either entity matches the trial, it will be instantly added to the provisional list for further categorization (Figure 4C). The list generated by these matching strategies is further filtered by user-specified conditions, such as clinical trial phase, recruiting status, trial center location, patient gender and age, with only trials that meet the requirements being preserved (Figure 4D).

The clinical trials that are matched and filtered are stored in MongoDB in JSON format, utilizing the GridFS technology for space reduction due to the large amount of clinical trial data associated with plenty of matching jobs. Users can perform secondary filtering based on metadata and entity data, retaining trials that meet the criteria and removing those that do not. The final filtered list is saved in the same format and can be re-screened by the user at any time to obtain a satisfactory list of clinical trials that meet their requirements in terms of both quality and quantity.

### System implementation

OncoCTMiner comprises a web application (APP) system and multiple application programming interfaces (APIs). The OncoCTMiner APP system was developed using SpringBoot (v2.3.1), Mybatis-plus (v3.3.2), LayUI (v2.5.6), EasyWeb (v3.1.8) and jQuery (v3.2.1). The OncoCTMiner APIs provide programming access to all clinical trial search functionalities in the BioC-JSON format. These APIs were written in Python and built using the Flask-RESTful framework. Database management for both the APP and APIs is supported by MySQL (v8.0.28) and MongoDB (v5.0.9).

## Results

### Entity tagging results

The eligibility database of OncoCTMiner currently includes 460 952 clinical trials, among which 122 706 are cancer-related or contain cancer terms, with 2227 studies receiving manual double review. These trials are categorized into six categories, comprising over 8.15 million entities and over 9.32 million entity-criteria-trial triplets. Among the recognized entities, ‘surgery’, ‘chemotherapy’ and ‘radiation therapy’ are the top three entities that appear in 131 050, 48 273 and 42 909 clinical studies, respectively. In terms of entity eligibility categorization, the entities most closely related to clinical trials in the inclusion criteria are ‘breast Cancer’, ‘non-small cell lung cancer’ and ‘solid tumor’, which are associated with 8268, 4909 and 3474 trials, respectively. The top three entities with the largest number of associated clinical trials in the exclusion criteria are ‘surgery’, ‘transplantation’ and ‘radiation therapy’. Drugs account for more than 42.86% of the entities that appear in at least three clinical trials under exclusion criteria. When therapies are considered, the proportion increases to 53.91%. It should be noted that some therapy entities that could be classified as exclusion criteria in part are judged to be non-criteria due to the presence of specific conditions. If these entities are included, this ratio will be significantly increased, highlighting the importance of prior treatment history (drugs or other types of therapies) in excluding unsuitable clinical trial candidates (for additional statistical results, please refer to https://oncoctminer.chosenmedinfo.com/assets/xlsx/statistics_on_eligibility_criteria_database.xlsx).

### Eligibility database

We created a precision oncology clinical trial eligibility database by identifying and standardizing biological concepts extracted from clinical trial data. The trial metadata and textual information are stored in the MongoDB database in BioC-JSON format, while the six categories of entities identified from the eligibility criteria, their corresponding standard entries and the eligibility criteria classification data are stored in a structured form in the MySQL database. This allows for easy querying and matching of clinical trials in the future.

### Trials searching

#### Quick search

**Figure 5.**
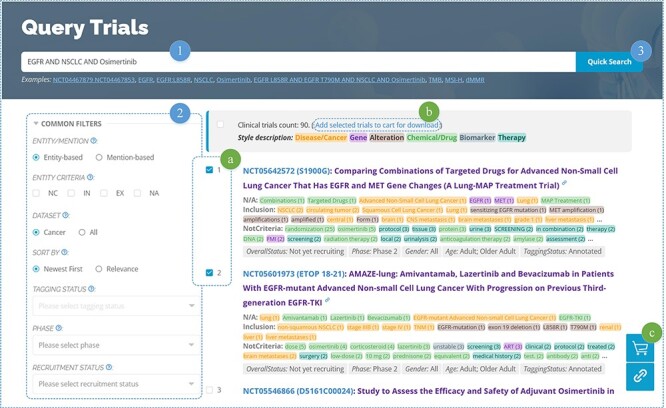
Clinical trials searching.

OncoCTMiner features a rapid search function (Figure 5–1), similar to many other databases. Users can enter keywords related to cancer type, genes, mutations, drugs/chemicals, biomarkers, therapies, clinical trial identifiers and more into a single input field, allowing for a comprehensive retrieval of clinical trials related to those keywords. By default, the system utilizes string searching to match the user’s keyword with the recognized entity (mention-based) and returns a match if found. In order to achieve more precise semantic matching, we also provide an entity-based matching method. After entering the keyword, the system searches for matching standard entries in the terminologies and returns all of them. The user selects the target entity and clicks on the entity link to search using the standardized entity, thereby retrieving all clinical trials associated with that entity.

1) Enter entity-related keyword(s), 2) set filters, and then 3) click the search button to display the search results. Additionally, users can (a) select different trials and (b) add them to their trials cart for subsequent download.

We not only enable entity-level retrieval of cancer clinical trials but also offer more accurate querying based on the criteria categorization information for each entity, which sets us apart from competing solutions. For instance, non-melanoma skin cancer is included in some clinical trials such as NCT00518037, meaning that patients with this type of cancer may consider participating in the trial, subject to other inclusion criteria. On the other hand, it may appear as an exclusion criterion in some clinical studies, such as NCT03581357, indicating that individuals with this condition are not eligible to participate. However, non-melanoma skin cancer or similar items are often found in the exclusion criteria of many clinical trials as exceptions to particular exclusion requirements, such as NCT04465942 and NCT04445844. It should be noted that this specific type of cancer is simply mentioned and neither used as an inclusion nor an exclusion criterion for the therapeutic trials being discussed.

#### Advanced search

In the era of precision medicine, searching based on biomedical concepts can be a valuable supplement to the query function of clinical trial metadata available on websites such as ClinicalTrials.gov. However, it cannot replace the conventional retrieval function based on metadata information, which can still be useful in screening clinical trials. For instance, in urgent situations, some patients may want to be recruited as soon as possible after identifying a suitable clinical trial. In such cases, they need to quickly eliminate clinical trials that have not yet started recruiting, have stopped recruiting, or have already finished. In this regard, clinical trial recruitment status information can help filter out unnecessary information, saving patients’ time. To enable users to perform entity-based and metadata-based combined retrieval operations in a single step, we also provide advanced search features (Figure 5–2) that allow users to combine conditions and conduct exact searches across the entire database.

### Sample-trials matching

**Figure 6.**
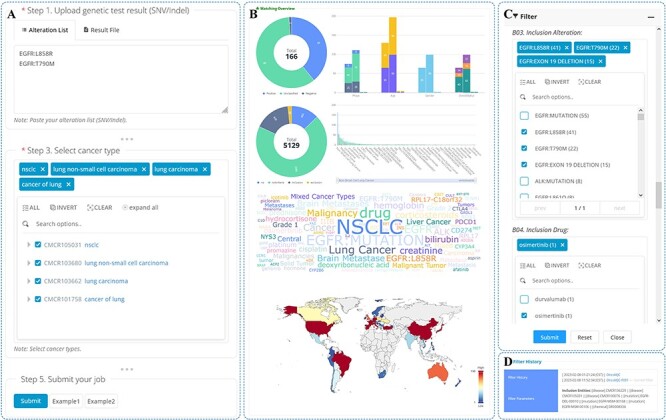
Trials matching.

The search functionality is limited in its ability to retrieve a large amount of information at once. To address this issue, OncoCTMiner includes a trial matching tool for batch quick retrieval of clinical trials at the individual level. Specifically, we have developed a patient-trial matching function that leverages tumor patients’ genetic testing results and cancer type information as the fundamental criteria for pre-screening precision oncology clinical trials (see Figure 6A).

An overview of the trial matching and pre-screening function is shown. A) On the trial matching page, users can enter genetic test results, select cancer types and submit a matching job. B) The matching report displays jobs that have been matched or filtered and provides a permanent URL for users to access the report at any time. C) Users can set filters on the matching report page to screen trials based on the list of matches. D) The filter history can be viewed from the matching report page.

We have made the process of utilizing OncoCTMiner’s trial matching tool simple and user-friendly. Users can either copy and paste the bioinformatics analysis results of the genetic testing data of tumor patient samples into the text field or directly upload them in VCF format. While the variation of single nucleotide variants (SNV) or short insertions/deletions (Indel) are the key variation types supported, OncoCTMiner also supports other types of alterations, including copy number variation (CNV), gene fusion and expression status. Users only need to input the gene list in the appropriate text area. Additionally, the system supports clinical trial matching of tumor TMB, MSI and MMR Along with the mutation test results, users must also provide the type of cancer by retrieving and selecting the appropriate cancer name from the cancer tree provided. Users may also include additional conditions in the matching step by selecting meta-information such as recruitment status, stage, age, gender, and the country where the trial unit is located.

After all parameters have been entered or selected, the user can submit the job, and the system will run the corresponding matching procedure in the background. Typically, tasks submitted by the user are completed within a reasonable amount of time, and a permanent uniform resource locator is provided for the user to access the matching report page at any time (as shown in Figure 6B and Figure 6C). Multiple matching jobs can be submitted by a user, and they can view them all on the job list page, with completed jobs having direct links to the corresponding report pages.

The matching report page provides all information related to the matching job. The ‘Job Details’ section displays basic information about the matching job, such as the submission time and the corresponding parameters. In the ‘Variant Annotation Results’ section, the variation list in the terminology that the system’s variant annotation procedure matched by the variants provided or uploaded by users is shown, and these serve as the conditions that are directly utilized for trial matching. The ‘Match Result Overview’ section provides an overview of the matched clinical trials in the form of various statistical graphs, such as the number of categories, meta-information distribution (e.g. stage and recruitment status), statistical distribution of entities contained in the trial and statistics of the countries or regions where they are located. Detailed information on all matched clinical trials is included in the ‘Matched Clinical Trials’ section.

### Trials filtering

The matching criteria used by OncoCTMiner can be quite broad, resulting in a large number of potential clinical trials being identified. To help users narrow down their search, OncoCTMiner provides a trial screening feature (as shown in Figure 6D) that enables more accurate refinement of the results. Users can refine their criteria based on the initial list of matches, for example, by selecting trials that require specific mutations or excluding therapies that have been found to be ineffective. Once the refined criteria are submitted, the system performs the necessary filtering operations in the background, generating a new report that is linked to the original report. The filtering can be further refined on either report, allowing users to gradually identify the most suitable clinical trials.

### Use guide

To facilitate a better understanding of the system’s capabilities, we offer a web-based user guide (the ‘TUTORIAL’ page) that covers all of the system’s main features, including step-by-step tutorials on clinical trial search and matching services as mentioned above. The corresponding examples are also available on the relevant function pages.

## Discussion

Precision oncology therapy has benefited many cancer patients after undergoing clinical validation. However, for continued advancement in the development of more effective treatments, more cancer patients need to participate in clinical trials to test novel precision cancer treatments. Despite genetic testing becoming more common among cancer patients, only a small proportion of those with actionable mutations participate in precision oncology clinical trials, estimated at 10–15%. There are various reasons for this poor clinical trial participation, including limited awareness among patients and physicians about relevant clinical trials, as well as a lack of sophisticated trial matching technology to automate patient-trial matching.

To address the issue of poor participation in precision oncology clinical trials, we developed OncoCTMiner, a precision oncology eligibility criteria database and trial matching system. Using natural language processing (NLP), we analyzed clinical trial textual data and created a database with human tagging and reviewing, providing users with a comprehensive and accessible search engine for precision oncology clinical trials. Our system matches the corresponding eligibility entities of precision oncology clinical trials based on cancer patients’ clinical data and omics alterations identified in samples, and then preliminarily categorizes the matched clinical trials based on the matching results and entity categorization criteria. Users can then perform further screening based on information such as clinical treatment history and trial recruitment status until they obtain an ideal qualified clinical trial list.

We recognize that OncoCTMiner has its limitations. While it is novel and efficient to identify bio-entities from clinical trial textual data and to determine the inclusion or exclusion criteria corresponding to each entity, enabling the construction of a precise oncology clinical trial eligibility database and a patient clinical trial matching platform, the efficiency and accuracy of biological entity identification can be influenced by existing NLP technology, preventing 100% precision. Nonetheless, the manual tagging platform and multiple review mechanisms we have established allow for the evaluation and amendment of NLP recognition results, resulting in a high-quality precision oncology clinical trial eligibility database that can achieve more accurate clinical trial matching over time. Additionally, we plan to include other entity categories, such as phenotypes, in the future. Phenotypes are often specified in eligibility criteria in addition to cancer types, genes, alterations, drugs and therapies. For example, a patient might only be included if a certain phenotypic condition is met, or if a specific phenotype occurs, the patient should be excluded from certain clinical trials.

## Conclusions

OncoCTMiner is a cutting-edge platform and knowledge base system for mining precision oncology clinical trial eligibility data, which provides fast and efficient information retrieval capabilities. The platform’s oncology trial pre-screening functionality can match clinical trials in real-time based on patients’ genetic profiles and clinical data, providing cancer patients with greater hope. This can lead to increased accrual rates for precision oncology clinical trials, speeding up the development of potential high-efficiency tumor treatments and benefiting more cancer patients.

## Supplementary Material

baad077_Supp

## Data Availability

OncoCTMiner is free and open to all users. OncoCTMiner can be accessed at https://oncoctminer.chosenmedinfo.com.
