# Supplementary material for: OncoCTMiner: streamlining precision oncology trial matching via molecular profile analysis
Source: Database (Oxford). 2023 Nov 4;2023:baad077. doi: 10.1093/database/baad077 (PMC10630409; doi:10.1093/database/baad077)
Supplement: baad077_Supp [file baad077_supp.zip › suppl_data/SupplementaryFile1.pdf]

Supplementary Table S1: Comparison of OncoCTMiner to other systems.

Features \ Systems	OncoCTMiner	My Cancer Genome[1]	OCTANE[2]	MatchMiner[3]
Terminologies / Ontologies				
Disease/Cancer	NCIt, MeSH, Disease Ontology, OncoTree	DiseaseOntology, NCIt, WHO, OncoTree, SNOMEDCT, UMLS	-	OncoTree
Gene	HGNC, NCBI-Gene	RefSeq, UTA	NCBI-Gene	-
Alteration	CIViC, CGI, PMKB, DoCM, OncoPDSS, ClinVar, COSMIC	HGVS, ISCN, UTA	-	-
Chemical/Drug	DrugCentral, ChEBI, ChEMBL, PharmGKB, DrugBank,	NCIt, NCIm, SNOMED CT, DrugBank, VANDF	NCIt	-
Biomarker	Self-built terminology	-	-	-
Therapy	Self-built terminology	-	-	-
Data Related				
Trials Source(s)	ClinicalTrials.gov	NCI-Supported Clinical Trials, UMIN Clinical Trials Registry, ClinicalTrials.gov	ClinicalTrials.gov, CORe	ClinicalTrials.gov
Trails Content	Eligibility criteria, Meta data, Other detailed info	Eligibility criteria, Meta data, Other detailed info	Eligibility criteria, Meta data, Other detailed	Eligibility criteria, Meta data
Entity-level criteria	Yes	Yes	-	Yes
Research Field	Cancer	Cancer	Cancer	Cancer
Clinical trials	460,952	9,809	5,439	-
Disease/Cancer	14,439	955	252	-
Gene	6,772	18,271 (gene + alteration)	779	-
Alteration	4,691		-	-
Chemical/Drug	13,749	2,861	1,453	-
Biomarker	8	-	-	-
Therapy	1,401	-	-	-
Trials Update	Monthly	Every two weeks	Daily	Daily
Data format	Structured, BioC-JSON based	Structured, webpage based	Structured，Oracle database	Structured, CTML based
Functions				
Manual tagging function	√	-	-	-
Trials searching	√	√	√	√
Entity-based	√	√	-	√
Metadata-based	√	√	-	√
Trials matching	√	-	√	√
Variant Annotation	√	-	-	-
VCF-supported	√	-	-	-
Patient-level	√	-	-	√
Trials Filtering	√	-	-	√
Trials Download	√	-	-	-
RESTful API	Yes, open and free	Yes, need license	-	-
Web-based open access	Yes	Yes	No	No
URL	https://oncoctminer.chosenmedinfo.com/	https://www.mycancergenome.org/	https://pct.mdanderson.org/octane/	https://matchminer.org/

(to be continued)

(continue)

DQueST[4]	Criteria2Query[5]	EliIE[6]	DCMS[7]
OMOP concepts	OMOP concepts	OMOP concepts, SNOMED-CT, LOINC	-
-	-	No	-
-	-	No	-
OMOP concepts	OMOP concepts	OMOP concepts	-
-	-	-	-
-	-	-	-
ClinicalTrials.gov	ClinicalTrials.gov criteria, user-entered criteria	ClinicalTrials.gov	MSKCC therapeutic clinical trials
Eligibility criteria	Eligibility criteria	Eligibility criteria	Eligibility criteria, Meta data, Other detailed info
-	-	-	-
Does not focus on specific field	Does not focus on specific field	Does not focus on specific field	Cancer
252,330	-	230 (training set)	840
-	-	-	Not mentioned
Not mentioned	-	-	Not mentioned
Not mentioned	-	-	Not mentioned
-	-	-	Not mentioned
-	-	-	-
-	-	-	-
Not mentioned	-	-	Daily
Not mentioned	Structured, JSON based	Structured, XML based	Structured
-	-	-	-
√	-	-	-
√	-	-	-
Yes, dynamic questionnaire	-	-	-
No	-	-	√
No	-	-	-
No	-	-	-
No	-	-	√
√	-	-	-
-	√	-	-
-	Yes	-	-
Website unavailable	Website unavailable	-	No
https://impact.dbmi.columbia.edu/dquest-flask/	http://www.ohdsi.org/web/criteria2query/	-	-

Note:

1 The comparative data of My Cancer Genome is derived from:

a) Holt, M.E., Mittendorf, K.F., LeNoue-Newton, M., Jain, N.M., Anderson, I., Lovly, C.M., Osterman, T., Micheel, C. and Levy, M. (2021) My Cancer Genome: Coevolution of Precision Oncology and a Molecular Oncology Knowledgebase. JCO Clin Cancer Inform, 5, 995-1004.

b) https://www.mycancergenome.org

2 The comparative data of OCTANE is derived from:

a) Zeng, J., Shufean, M.A., Khotskaya, Y., Yang, D., Kahle, M., Johnson, A., Holla, V., Sanchez, N., Mills Shaw, K.R., Bernstam, E.V. et al. (2019) OCTANE: Oncology Clinical Trial Annotation Engine. JCO Clin Cancer Inform, 3, 1-11.

b) https://pct.mdanderson.org

3 The comparative data of MatchMiner is derived from:

a) Klein, H., Mazor, T., Siegel, E., Trukhanov, P., Ovalle, A., Vecchio Fitz, C.D., Zwiesler, Z., Kumari, P., Van Der Veen, B., Marriott, E. et al. (2022) MatchMiner: an open-source platform for cancer precision medicine. NPJ Precis Oncol, 6, 69.

b) https://matchminer.org

4 The comparative data of DQueST is derived from:

a) Liu, C., Yuan, C., Butler, A.M., Carvajal, R.D., Li, Z.R., Ta, C.N. and Weng, C. (2019) DQueST: dynamic questionnaire for search of clinical trials. J Am Med Inform Assoc, 26, 1333-1343.

b) https://impact.dbmi.columbia.edu/dquest-flask

5 The comparative data of Criteria2Query is derived from:

a) Yuan, C., Ryan, P.B., Ta, C., Guo, Y., Li, Z., Hardin, J., Makadia, R., Jin, P., Shang, N., Kang, T. et al. (2019) Criteria2Query: a natural language interface to clinical databases for cohort definition. J Am Med Inform Assoc, 26, 294-305.

b) http://www.ohdsi.org/web/criteria2query

6 The comparative data of EliIE is derived from:

a) Kang, T., Zhang, S., Tang, Y., Hruby, G.W., Rusanov, A., Elhadad, N. and Weng, C. (2017) EliIE: An open-source information extraction system for clinical trial eligibility criteria. J Am Med Inform Assoc, 24, 1062-1071.

7 The comparative data of DCMS is derived from:

a) Eubank, M.H., Hyman, D.M., Kanakamedala, A.D., Gardos, S.M., Wills, J.M. and Stetson, P.D. (2016) Automated eligibility screening and monitoring for genotype-driven precision oncology trials. J Am Med Inform Assoc, 23, 777-781.

* The '-' in each cell indicates that the information is not availeble or that the feature does not exist.
